# Ultrathin TiO_2_–CeO_2_ Composite Films for Efficient Photocatalytic Methylene Blue Degradation

**DOI:** 10.1021/acsomega.6c07126

**Published:** 2026-09-17

**Authors:** Mahsa Amiri, Majid Ahmadi, Mahtab Salari Mehr, Elyad Damerchi, Khatereh Roohi, Peyman Taheri, Tanel Tätte, Nadezda Kongi, Alexander Vanetsev

**Affiliations:** † Institute of Physics, 37546 University of Tartu, W. Ostwaldi 1, 50411 Tartu, Estonia; ‡ Faculty of Science and Engineering, University of Groningen, 4 Nijenborgh, 9747 AG Groningen, Netherlands; § Institute of Technology, University of Tartu, Nooruse 1, 50411 Tartu, Estonia; ∥ Department of Materials Science and Engineering, Delft University of Technology, 2 Mekelweg, 2628 CD Delft, Netherlands; ⊥ Institute of Chemistry, University of Tartu, Ravila 14A, 50411 Tartu, Estonia

## Abstract

Contamination of natural water resources by industrial organic pollutants poses serious environmental and health risks, creating a need for efficient and sustainable wastewater treatment technologies. TiO_2_ is widely regarded as an eco-friendly and low-cost photocatalyst, but it is normally used as a dispersed powder, which must be separated from the treated water. Immobilizing it as a thin film removes that step at the cost of a much smaller accessible surface, so the performance of such films depends strongly on their composition. In this study, ultrathin TiO_2_–CeO_2_ films containing 1, 2, and 3 wt % of CeO_2_ were prepared by sol–gel dip coating, and the influence of CeO_2_ content on their structure and photocatalytic performance was investigated. Electron microscopy and elemental mapping show distributed cerium, consistent with a TiO_2_–CeO_2_ nanocomposite. The degradation of methylene blue under UVA irradiation was used as a model reaction. The first-order rate constant passes through a maximum at 2 wt % CeO_2_, rising from 4.9 × 10^–3^ min^–1^ for pristine TiO_2_ films to 9.1 × 10^–3^ min^–1^, so that the ∼30 nm smooth film approaches the performance of *Degussa P25* suspended nanopowder at equal catalyst loading. The TiO_2_–CeO_2_(2%) film retains its photoactivity over four consecutive cycles. These results show that sol–gel TiO_2_–CeO_2_ thin films are efficient and reusable immobilized photocatalysts for water purification.

## Graphical abstract

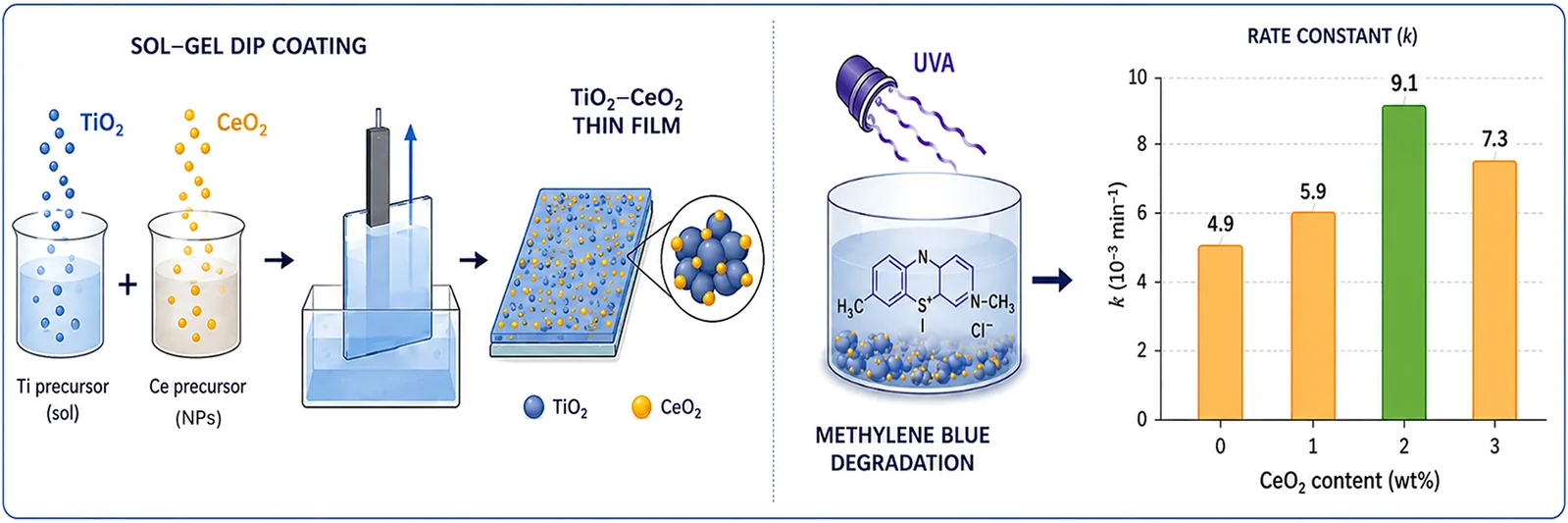

## 1 Introduction

Industrial wastewater frequently contains elevated levels of organic pollutants with long-lasting adverse effects on ecosystems and human health,^1^ creating a pressing demand for cost-effective and scalable technologies that remove such contaminants or convert them into less hazardous compounds. Photocatalytic degradation, based on environmentally benign redox reactions, is a promising route to this end.^2,3^ The process involves adsorption of the contaminant on the catalyst surface, generation of electron–hole pairs by light of energy *h*ν ≥ Eg, and redox decomposition of the adsorbed species by the resulting reactive oxygen species (ROS).^4,5^ Its efficiency is limited chiefly by insufficient charge-carrier generation and rapid electron–hole recombination, and depends further on the catalyst properties, the nature and concentration of the contaminant, the solution pH, and the irradiation conditions.^6^ Recent work has therefore targeted heterojunction formation, surface engineering, impurity doping, and composites with conductive carbon or polymer phases.^7,8^

Dyes such as methylene blue (MB) are widely used as model compounds in these studies, since their concentration is readily quantified by visible-range spectroscopy and many of them, MB included, are toxic and mutagenic, making their removal from textile, paper, and food-industry effluent an environmental priority.^9–11^ Advanced oxidation processes have accordingly been reviewed extensively as sustainable routes for degrading MB-laden textile effluent, with photocatalysis favored for its low cost and mild operating requirements.^12^

Metal-oxide semiconductor catalysts can promote the oxidation of organic compounds in both air and water, leading to the mineralization of pollutants into water, CO_2_, and inorganic salts,^13,14^ and many have been examined for photocatalytic applications.^15–17^ Among them, TiO_2_ is the most extensively studied, owing to its high stability, low cost, and strong activity under UV irradiation.^18,19^ Its wide band gap of ∼3.2 eV,^20,21^ however, restricts sunlight absorption, and across all of its applications, from photocatalysis and dye-sensitized solar cells to gas sensing and biomedical coatings, performance is curtailed by the same two intrinsic limitations: weak visible-light absorption and rapid electron–hole recombination.

Doping is a key strategy for engineering the band gap and photocatalytic activity of TiO_2_, modifying its electronic structure through defect states that lower the band gap energy. Blanco-Vega et al.^21^ narrowed the gap of microwave-assisted sol–gel TiO_2_ from 3.08 eV (the value reported for the pristine sample of that study) to 2.72 eV by Ni^2+^ doping, raising bisphenol A removal from 60 to 93% after 120 min. Al, Cu, Mo, and W act similarly: Al^3+^ substitution generates oxygen vacancies that increase photocatalytic activity, with Al and Cu reaching 70% and Mo and W 96% degradation under 60 min of visible-light irradiation.^22^ More broadly, the interplay between substitutional impurities and the annealing environment provides a general handle on the defect chemistry of solution-processed oxide semiconductors.^23^ For example, in solution-derived Co- and Cu-*co*-doped ZnO films, both the oxygen-vacancy concentration and the resulting defect-related emission and band gap narrowing were strongly influenced by the dopant concentration and by whether the films were annealed in air or argon.^24^ The principle extends beyond oxides: in solution-processed tin sulfide films, the choice of tin precursor alone determines whether p-type SnS or n-type SnS_2_ is obtained,^25^ establishing precursor chemistry, dopant level, and annealing environment as general control parameters over defect chemistry and charge-carrier behavior in solution-derived semiconductor films.

Among the various routes available for depositing metal-oxide films, the physical and vapor-phase techniques such as magnetron sputtering, chemical vapor deposition, pulsed laser deposition, and atomic layer deposition, deliver excellent thickness uniformity and interface abruptness, but they rely on vacuum systems, expensive instrumentation, and frequently elevated substrate temperatures, and they afford little latitude for introducing preformed secondary nanophases during growth.^26,27^ Solution-based sol–gel processing sidesteps these constraints, as it operates at low temperature and ambient pressure, uses inexpensive precursors, and permits the composition to be tuned simply by adjusting the sol chemistry.^26,28^ Even subpercent changes in composition can reshape film morphology and electrical response. When paired with dip-coating, it becomes especially well-suited to depositing uniform, ultra**-**thin layers over large substrate areas at minimal cost, and it readily accommodates the dispersion of discrete nanoparticles within the growing oxide network; a combination that has been exploited to produce photocatalytically active oxide films such as sol–gel dip coated CuO for dye degradation.^27^ A further practical advantage of the immobilized thin-film geometry is that it eliminates the filtration and centrifugation steps required to recover suspended powder catalysts, thereby simplifying catalyst reuse and making such coatings well suited for continuous-flow water-treatment applications.^29^

These processing advantages are most valuable when the film chemistry itself is engineered for efficient photocatalysis, and the TiO_2_–CeO_2_ system is well matched to this purpose. CeO_2_ was among the first materials studied in combination with TiO_2_, and TiO_2_–CeO_2_ nanocomposites were found to have a significantly narrower optical band gap than pure TiO_2_.^30^ Cerium oxide combines a high oxygen storage-and-release capacity with facile redox switching between Ce^3+^ and Ce^4+^. and this redox couple,^31^ together with the accompanying oxygen-vacancy network, enables its usefulness as a photocatalytic modifier. The vacancies and Ce^3+^/Ce^4+^ transitions provide intragap trap states that intercept photogenerated electrons, suppress electron–hole recombination, and thereby extend carrier lifetimes and increase reactive-oxygen-species generation.^9^ Incorporating CeO_2_ into a TiO_2_ matrix therefore offers a route to defect engineering and light absorption range modulation that pristine TiO_2_ films cannot access on their own.^32^ When the cerium oxide is introduced as preformed crystalline particles rather than as a soluble precursor, this approach is distinct from lattice doping and is expected to yield a TiO_2_–CeO_2_ nanocomposite in which the active chemistry resides at the interfaces between the two oxides. We previously reported an ultrathin, highly defective sol–gel TiO_2_ film that showed good photoelectrocatalytic efficiency for CO_2_ reduction but was limited by low electrical conductivity and the need for UV activation.^33^ In the present study, 2–5 nm CeO_2_ particles were incorporated into sol–gel TiO_2_ films deposited by dip-coating, and their influence on the photocatalytic behavior was examined. Relative to previously reported TiO_2_-based photocatalytic systems, these ultrathin TiO_2_–CeO_2_ films are intended to serve as durable, reusable coatings for water purification rather than dispersed powders, which cannot be recovered quantitatively from the treated water after use, a limitation that becomes more severe as particle size decreases into the nanometer range.^34^

## 2 Materials and Methods

### 2.1 Thin Film Deposition

TiO_2_ alkoxide sol precursor was prepared by mixing titanium­(IV) butoxide (Ti­(OC_4_H_9_)_4_, Acros Organics, 99%) and 1-butanol (Sigma-Aldrich, 99.8%) in a 1:18 molar ratio using a magnetic stirrer. Mixing was applied for 15 min until visual homogeneity was achieved. Then, 5 mL of glacial acetic acid (CH_3_COOH, ≥99%) was added to the mixture as a polycondensation rate-controlling agent.^35^

After that, the mixture was stirred for 24 h to complete hydrolysis and formation of a stable sol, usable in further experiments.

Silicon wafers, used as film substrates, were prepared for deposition using the RCA (Radio Corporation of America) standard cleaning method,^36^ involving a sequential wash in a mixture of ammonia, hydrogen peroxide, and water, followed by rinsing in deionized water.

For the preparation of TiO_2_–CeO_2_ composite films, CeO_2_ nanoparticles of 2–5 nm in size, obtained in accordance with the previously published protocol,^33^ were dispersed in acetic acid. To ensure uniform mixing and deagglomeration of particles, the mixture was sonicated for 30 min. The mixture was then added to the vigorously stirred sol. CeO_2_ nanoparticles were added to the titania sol at concentrations of 1, 2, and 3 wt %. After the addition of CeO_2_ particles, the precursors were aged for 1 day before being used for dip coating.

The obtained sol precursors were deposited on silicon wafers using the dip coating technique. Each substrate was immersed in the sol for 60 s prior to withdrawal to ensure proper wetting. To obtain homogeneous thin films, substrates were immersed in and withdrawn from the sol at a constant speed of 2.0 cm min^–1^. The obtained fresh films were dried at 100 °C for 10 min. Calcination of films was done in a furnace in an Ar atmosphere to prevent undesired oxidation, at 550 °C for 2 h, applying 5 °C min^–1^ and 20 °C min^–1^ heating and cooling rates, respectively.

Powders were prepared by evaporating the solvent from the prepared sol precursors. After solvent evaporation, the gels were left for aging in ambient air conditions, and afterward were ground. Obtained powders were subjected to heat treatment at 550 °C, in a similar way to the heat treatment of thin film samples. The procedure yielded pristine TiO_2_ powders as well as TiO_2_–CeO_2_ materials with 1, 2, and 3 wt % CeO_2_. UV–vis Diffuse Reflectance Spectroscopy (DRS) was performed on the obtained powders.

### 2.2 Characterization

Surface morphology of the films was studied using scanning electron microscopy (SEM, FEI, Nanosem 450). Focused ion beam (FIB) sample preparation was performed using the FEI Helios G5 CX dual-beam SEM-FIB. The electron-transparent samples were prepared by depositing protective electron-beam-induced deposition (EBID) carbon and platinum layers on the film before ion beam-induced deposition (IBID) Pt protective layer. Cross-sectional chunks (15 × 2.0 × 5 μm^3^) were transferred to a copper half-grid and thinned down to 80–100 nm thickness using standard Ga-beam processing at 30 kV with a specific window (6.0 × 5.0 μm^2^). The remaining chunk was left thick to maintain rigidity and minimize bending and stress release in the e-transparent window. Final cleaning was done using low kV steps (5 and 2 kV) to clean the side surfaces of the lamellae. The FIB-prepared sample was examined using a double-corrected and monochromated Themis Z scanning/transmission electron microscope (S/TEM) operating at 300 kV in high-angle annular dark-field (HAADF) STEM and TEM modes. The beam convergence angle (in STEM mode) was measured at ∼24.5 mrad, and HAADF STEM images were captured with collection angles from 26 to ∼200 mrad. The TEM images were captured using a Ceta 16 M camera. Grain size distributions of the samples were quantitatively evaluated using ImageJ software.

X-ray photoelectron spectroscopic studies were conducted using an XPS spectrometer (PHI-TFA), at a vacuum of 10^–9^ mbar. The analyzed area was 0.4 mm in diameter, and the analysis depth was 3–5 nm. Narrow multiplex scans of the peaks were captured using a pass energy of 71.55 eV and a step size of 0.1 eV, employing a takeoff angle of 45° relative to the sample surface. Spectra were processed using Multipak v 8.0 to determine the elemental composition from the XPS survey spectra. Data acquisition and analysis were done using Augerscan Version 3.3.2, RBD Instruments, Inc. X-ray diffraction (XRD) studies were performed to gain information on the crystallography of the prepared samples using a Bruker D8 Advance diffractometer with Ni-filtered Cu Kα radiation.

Photocatalytic activity of TiO_2_ was evaluated, measuring degradation of methylene blue in water solutions under UV irradiation. Thin films (5 × 5 cm^2^, ∼30 nm thickness) on substrates were placed in a batch glass container, used as a reactor, filled with 30 mL of a 10 mg L^–1^ aqueous MB solution (Merck, ≥ 95%). To ensure stability in environmental conditions, the container was placed inside a moisture-controlled chamber throughout the experiment.

Prior to UV exposure, the samples were stored for 20 min in the dark to allow MB adsorption–desorption equilibrium on the film surface. A 0.5 cm long magnetic stirring bar was introduced into the reactor to maintain homogeneous mixing, with stirring at 150 rpm continued throughout the photocatalytic test. Illumination was provided by a self-constructed device, delivering 2.4–3.0 W m^–2^ intensity in the 315–400 nm UVA spectral range. Light intensity was measured at the sample height using a Delta Ohm UVA probe through borosilicate glass. At time zero and every 45 min thereafter, 3 mL aliquots of MB solution were withdrawn from the reactor and transferred to a 10 mm optical path length cuvette for optical light absorbance measurements. The UV–Vis absorbance of each aliquot was measured using a spectrophotometer (Agilent Cary UV–Vis-NIR 5000, Agilent Technologies, USA), corresponding to the characteristic absorbance peak of MB. To avoid the effect of lab light on results, the measurements were carried out in darkness. The aliquot solution was injected back into the reactor after the measurement. The photocatalytic performance of both pristine TiO_2_ and TiO_2_–CeO_2_ films was assessed by measuring the methylene blue degradation efficiency over time. Samples including pure TiO_2_ as well as TiO_2_–CeO_2_ were irradiated under UV light, with MB concentration changes monitored to determine degradation rates and overall efficiencies. UV–Vis absorbance spectra were recorded at specific time intervals (initial, 45, 90, 135, and 180 min) under UV irradiation for each sample. To evaluate reusability, the best-performing TiO_2_–CeO_2_(2%) film was additionally subjected to four consecutive degradation cycles under identical conditions, using a fresh MB solution for each cycle. The MB absorbance was recorded at 0, 90, and 180 min of each cycle, and the film was rinsed with deionized water and dried between cycles.

To benchmark the performance of TiO_2_–CeO_2_ films, widely used *Degussa P25* TiO_2_ powders were tested under identical conditions. The mass of *Degussa* powder was adjusted to match the TiO_2_ content in the thin films. Assuming that TiO_2_ in the form of anatase has a density of 3.890 g cm^–3^ and taking into account film dimensions (5 × 5 cm^2^, 30 nm thick), the equivalent amount of *Degussa* powder was calculated to be 0.3 mg. Thus, the 0.01 g L^–1^ *Degussa* water solution was prepared for this reason and described as TiO_2_ films.

## 3 Results and Discussion

### 3.1 Phase Composition and Surface Morphology of Sol–Gel Prepared TiO_2_ and TiO_2_–CeO_2_ Films

When metal alkoxides react with water, they undergo polycondensation reactions in accordance with the micelle-templated self-assembly of ligands (MTSAL) theory. The reaction mechanism is supported by the fact that at the very beginning of the process, metal oxide nanoparticles form, followed by particle growth. Finally, particles agglomerate as the stabilizing alkoxy layer is inhibited from their surface. Formation of homogeneous sols, suitable as precursors for film preparation as well as for fiber drawing, is supported by proper rheological behavior.^37^ Post processing of sol–gel prepared materials includes aging and thermal treatment steps according to the process described in a previously published report.^38^ During the aging, the low-molecular-weight species and excess solvent are removed from the precursor network, leading to material densification. During the thermal treatment of sol–gel TiO_2_, residuals of organic matter remain. These residual organic species may remain trapped in the structure and carbonize at 250–400 °C. However, at temperatures above 400 °C, carbon oxidizes and materials start crystallizing.^37,39^

In our previous work on films prepared by this same sol–gel dip-coating route, TEM showed the obtained TiO_2_ film was crystalline, with interplanar spacing on crystallites corresponding to the (101) plane of the anatase TiO_2_ phase with an average film thickness of 30 nm. The STEM images showed that the pure TiO_2_ film exhibited a homogeneous and relatively smooth surface. The film appears dense and continuous, consistent with a well-formed nanocrystalline TiO_2_ thin film, as presented in our previous publication.^33^ In contrast, in the current study, the TiO_2_–CeO_2_ film reveals a distinctly different microstructure (Figure 1). The grain- and pore-size distributions for pristine and TiO_2_–CeO_2_ films are shown in Figure 1. This analysis reveals two systematic and opposing trends. The mean grain size decreases markedly upon CeO_2_ incorporation, from 22.4 ± 0.4 nm for the pristine film to 12.5 ± 0.4 nm for the TiO_2_–CeO_2_(2%) film, while the mean pore size increases in the opposite direction, from 7.7 ± 0.5 nm to 17.8 ± 0.7 nm. A combination consistent with the literature data showing that the fine particles of the second phase tend to segregate at the grain boundaries of the main phase, pin them, and thereby restrict grain growth^40,41^ while opening up voids between the resulting grain clusters.

**Figure 1:**
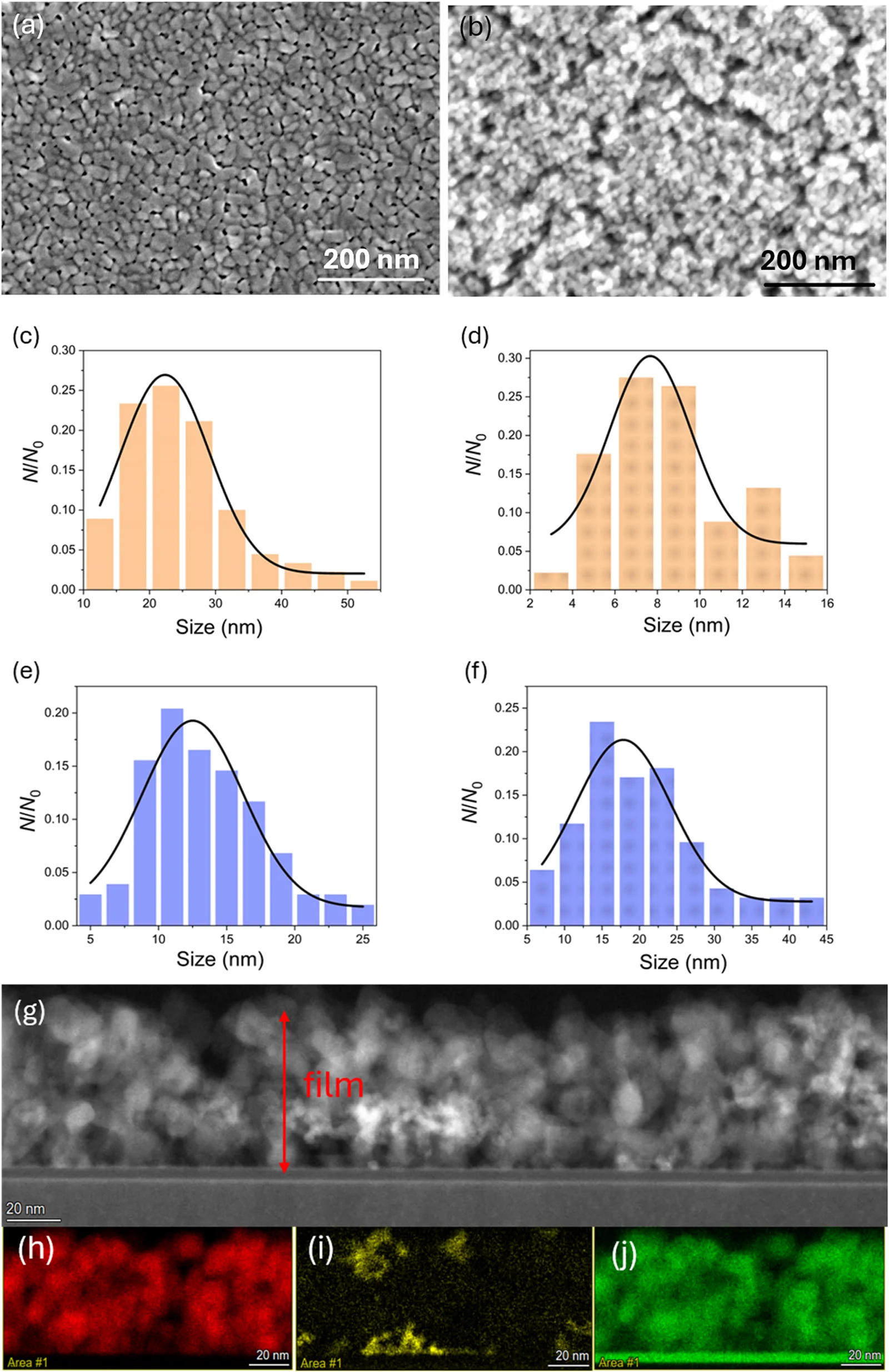
SEM images of the surface: (a) pristine TiO_2_ and (b) TiO_2_–CeO_2_ (2%) film. Grain- and pore-size distributions of (c, d) pure TiO_2_ and (e, f) the TiO_2_–CeO_2_ (2%) film, respectively. (g) Cross-sectional STEM image of the TiO_2_–CeO_2_ (2%) film with elemental distribution maps for: (h) Ti, (i) Ce, and (j) O.

The cross-sectional HAADF-STEM image of the TiO_2_–CeO_2_ (2%) film (Figure 1g) reveals an open, granular microstructure built from loosely packed nanoscale grains, with pronounced voids between grain clusters rather than a compact, continuous layer. This appearance is consistent with the quantitative analysis above: relative to the pristine film, the modified film combines finer grains with markedly larger intergrain pores, and the cross-section makes this enhanced open porosity directly visible. Such a morphology is expected to benefit photocatalytic performance, since the more open pore network improves accessibility of the internal film volume to the dye solution.^41,42^

The spatial distribution of cerium in this modified film was established by HAADF-STEM EDX elemental mapping (Figure 1h,i,j). Given that CeO_2_ was introduced as preformed 2–5 nm nanoparticles, and that the heat treatment at 550 °C is too low to drive solid-state diffusion of Ce into the anatase lattice, extensive substitutional or interstitial incorporation of Ce is not anticipated. The distribution signal from cerium atoms (Figure 1i) allows us to suggest that CeO_2_ nanoparticles are mainly concentrating on the outer surfaces of the film, both the open and film–substrate interface. The mapping is thus most consistent with a TiO_2_–CeO_2_ nanocomposite characterized by a high density of TiO_2_–CeO_2_ nanointerfaces, in which CeO_2_ is distributed but not dissolved in the TiO_2_ matrix. Accordingly, the effects analyzed in the following sections are attributed to the extended TiO_2_–CeO_2_ interface rather than to lattice-substitutional doping.^32^

The phase composition of the pristine TiO_2_ powder was examined by X-ray diffraction (Figure S1). Thirteen reflections are resolved and indexed between 20 and 90° 2θ, all consistent with the tetragonal anatase structure (space group *I*4_1_/*amd*) and matching the reported anatase reflection positions.^43^ No rutile (110 at 27.45° 2θ) or brookite (121 at 30.81° 2θ) reflections appear above the background, identifying anatase as the only detectable crystalline phase and supporting the anatase lattice fringes previously resolved by TEM for films prepared by the same route.^33^ For TiO_2_–CeO_2_ samples, the low cerium loading (≤3 wt %) and the 2–5 nm size of the CeO_2_ nanoparticles place the CeO_2_ reflections close to the detection limit of laboratory X-ray diffraction. For that sample we therefore focus on HAADF-STEM imaging, EDX elemental mapping, and XPS.

X-ray Photoelectron Spectroscopy was conducted to study the chemical state of the TiO_2_–CeO_2_ film with 2 wt % CeO_2_ (Figure 2). The sample was selected because of its highest efficiency in terms of MB degradation rate in the methylene blue degradation experiments, which will be discussed in Section 3.3.

**Figure 2:**
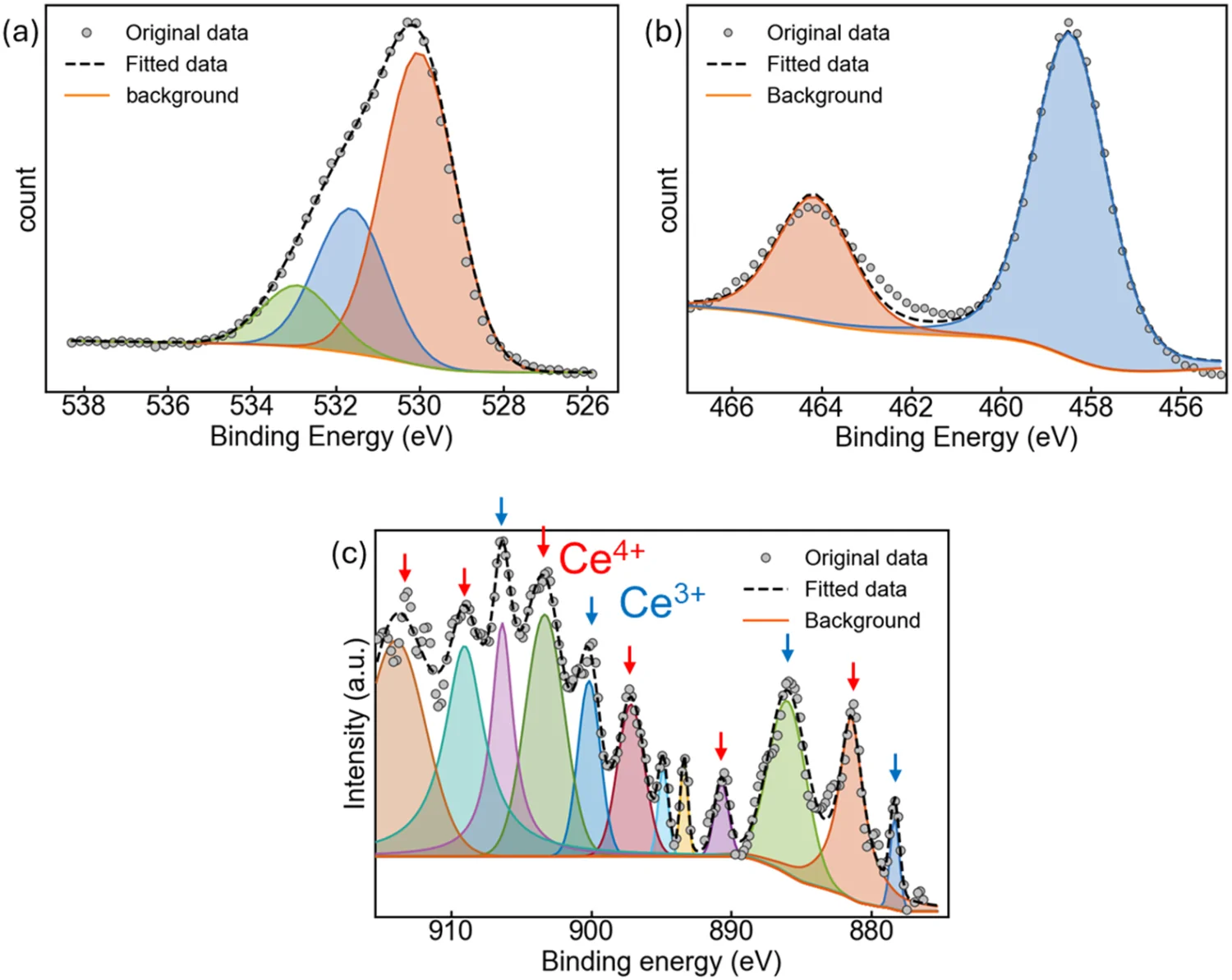
XPS spectra of (a) Ti 2p, (b) O 1s, and (c) Ce 3d core levels for TiO_2_–CeO_2_(2%) thin film.

In our previous work,^33^ using the same thin-film preparation method, the Ti 2p spectrum of pure TiO_2_ exhibited the characteristic Ti^4+^ doublet together with a weak Ti^3+^ component, indicating the presence of oxygen-vacancy-related titanium reduction defects. The formation of Ti^3+^ species was attributed to oxygen vacancies within the TiO_2_ lattice, which introduce defect states that can modify the electronic structure and influence the photocatalytic properties of the film.^44^

In contrast, the Ti 2p spectrum of the TiO_2_–CeO_2_(2%) thin film (as shown in Figure 2a) is fitted using only the Ti^4+^ doublet, with the Ti 2p_3/2_ and Ti 2p_1/2_ peaks located at approximately 458.55 and 464.32 eV, respectively (Δ*E* = 5.77 eV). These binding energies are in good agreement with previously reported high-resolution XPS data for Ti^4+^ in TiO_2_.^45^ No Ti^3+^ component is resolvable in the TiO_2_–CeO_2_ film. Cerium-associated oxygen defects may nevertheless be present, owing to the reversible Ce^3+^/Ce^4+^ redox couple, and CeO_2_ can introduce its own oxygen-vacancy-related defect states, which are reflected in the O 1s spectra discussed below.^46,47^

The O 1s XPS spectra of both pristine TiO_2_ and TiO_2_–CeO_2_(2%) provide insights into the oxygen environment and defect states. For the pure TiO_2_ samples, the deconvoluted O 1s peaks appear at approximately 529.71, 531.7, and 533.04 eV, corresponding to lattice oxygen (O_L_), oxygen vacancies (O_V_), and adsorbed oxygen species (O_A_), respectively.^44^ In the TiO_2_–CeO_2_(2%) film, the O 1s spectrum shows a similar three-component structure; however, the relative intensity of the intermediate component at ∼531–532 eV is increased. This increase is consistent with a contribution from Ce–O bonds. No Ti^3+^component is resolvable in the Ti 2p spectrum of the same film.

The Ce 3d spectra of the TiO_2_–CeO_2_(2%) film provide insight into the oxidation states and electronic interactions between CeO_2_ and TiO_2_. The deconvoluted Ce 3d spectrum reveals multiple overlapping peaks over the entire measured binding energy range, characteristic of cerium in mixed oxidation states within the TiO_2_–CeO_2_ matrix. The complexity of the Ce 3d spectral envelope, arising from multiple splitting and final state effects inherent to cerium compounds, is consistent with the coexistence of Ce^3+^ and Ce^4+^ oxidation states.^48,49^ The fitted components indicate the presence of both Ce^3+^ and Ce^4+^-related features, as shown in Figure 2. Owing to the relatively low CeO_2_ concentration (2 wt %), the Ce signal is weak; therefore, the Ce 3d spectrum is interpreted qualitatively, and no quantitative Ce^3+^/Ce^4+^ ratio is reported. These findings indicate that cerium is present in mixed valence in the TiO_2_–CeO_2_(2%) film.

### 3.2 UV–vis Diffuse Reflectance Spectroscopy Analysis

Since the obtained sol–gel TiO_2_ films were too thin for optical measurements,^50,51^ powders were prepared using the same synthesis protocol as that used for the thin films, thereby producing materials with a comparable structure. Diffuse reflectance spectroscopy (DRS) is optimized for bulk or powder samples where light can scatter within the material, providing a robust absorption profile.^52,53^

The DRS spectra of pristine TiO_2_ and the 1, 2, and 3 wt % CeO_2_ powders are shown in Figure 3a. The pristine TiO_2_ samples exhibited a sharp absorption edge around 400–410 nm, typical of anatase at a slightly longer wavelength than expected for the canonical anatase value of ∼3.2 eV^22^ which is attributed to the defects originating from sol–gel synthesis. With increasing of CeO_2_ content, the absorption edge shifts to longer wavelengths and the subedge absorption increases. As the two oxides are present as separate phases, this additional absorption is assigned to the CeO_2_ phase rather than to a modification of the TiO_2_ absorption edge.

**Figure 3:**
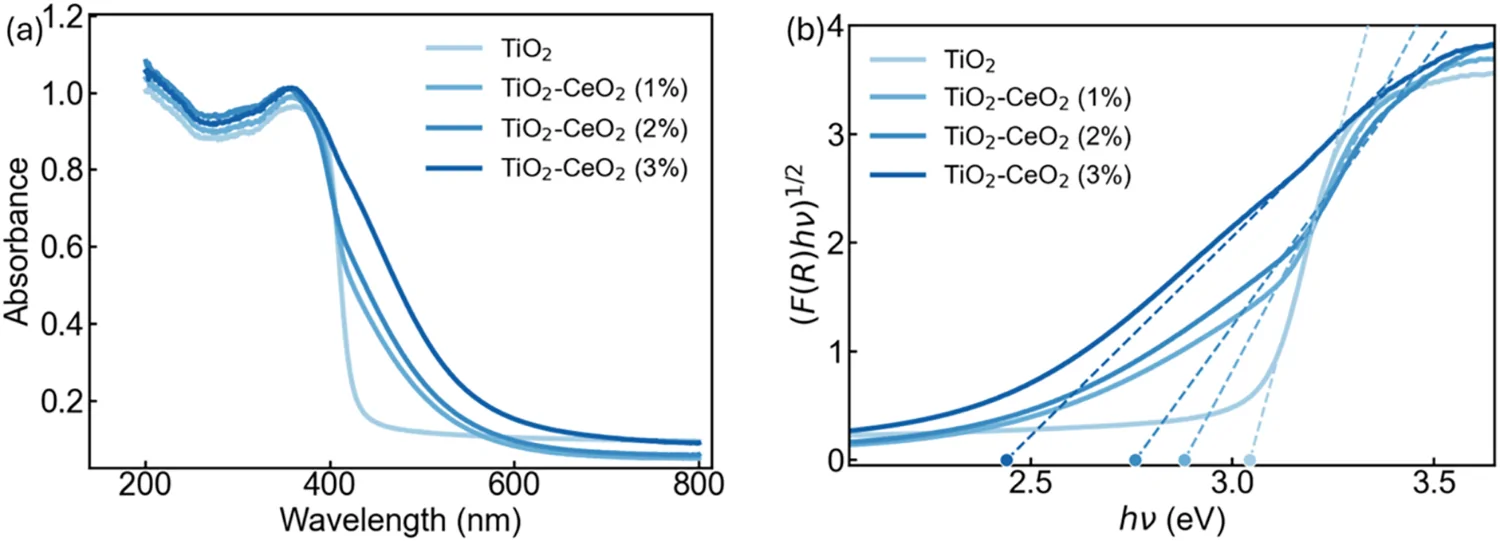
UV–vis diffuse reflectance spectra (DRS) (a) and corresponding Tauc plots (b) of the as-prepared powders.

To quantify the energy gaps of prepared materials, the Tauc plot method was employed. The obtained results are presented in Figure 3b. The calculation formula for energy gap determination is given as

1 $${\left(\alpha h\nu \right)}^{n}=A\left(h\nu -E_{g}\right)$$

Here, α, *h*, ν, and *E*_g_ represent the absorption coefficient, Planck constant, optical frequency, and band gap energy, respectively, and *A* is a constant related to the material properties.^54^

The exponent *n* characterizes the nature of the interband transition: *n* = 2 for direct allowed and *n* = 1/2 for indirect allowed transitions.^55^ Anatase TiO_2_ possesses an indirect fundamental gap, with its valence band maximum and conduction band minimum at different points in the Brillouin zone, so that the lowest energy interband transition is phonon assisted and indirect.^56^ The gaps were evaluated from indirect Tauc plots of (*F*(*R*) *hν*)^1/2^ against hν, where *F*(*R*) is the Kubelka–Munk function of the measured diffuse reflectance, used in place of α by extrapolating the tangent of the linear edge region to (*F*(*R*)­hν)^(1/2)^ = 0 (Figure 3b). The values obtained are 3.1 eV for pristine TiO_2_, 2.9 eV at 1 wt %, 2.8 eV at 2 wt %, and 2.5 eV at 3 wt %. These values are reported as apparent band gaps. The measured absorption of a two-phase material is the sum of the TiO_2_ and CeO_2_ contributions, whose onsets overlap and are broadened by defect states in both phases. At 1–3 wt % CeO_2_ the response is dominated by TiO_2_. The apparent gap therefore describes the powder as a whole rather than either phase individually. The subgap absorption tail becomes more pronounced with the increase of the CeO_2_ content, as it may be expected.^57,58^

### 3.3 Photocatalytic Activity Test

MB light absorption has the peak absorbance at 664 nm, according to the previously published reports.^59–61^ In our experiments, the intensity of this band decreased gradually for the pristine TiO_2_ film (Figure 4b), indicating moderate degradation, whereas the TiO_2_–CeO_2_(2%) film showed the fastest decrease (Figure 4d). The TiO_2_–CeO_2_ films with 1 and 3 wt % CeO_2_ demonstrate intermediate performance. Notably, the TiO_2_–CeO_2_(2%) thin film exhibited photocatalytic performance comparable to that of *Degussa P25* TiO_2_ powder (Figure 4). However, it is important to highlight that while *Degussa* consists of nanostructured particles offering a high surface area for contact with the dye solution, the synthesized films present a much more limited reactive interface restricted to a single exposed planar surface. Achieving comparable degradation efficiency despite these geometrical constraints demonstrates that the thin-film architecture, together with CeO_2_ incorporation, provides an effective photocatalytic configuration. This result further suggests that engineered thin films can serve as effective, reusable photocatalysts in practical applications where compact design and quick and efficient separation of catalyst from the reaction mixture are advantageous.

**Figure 4:**
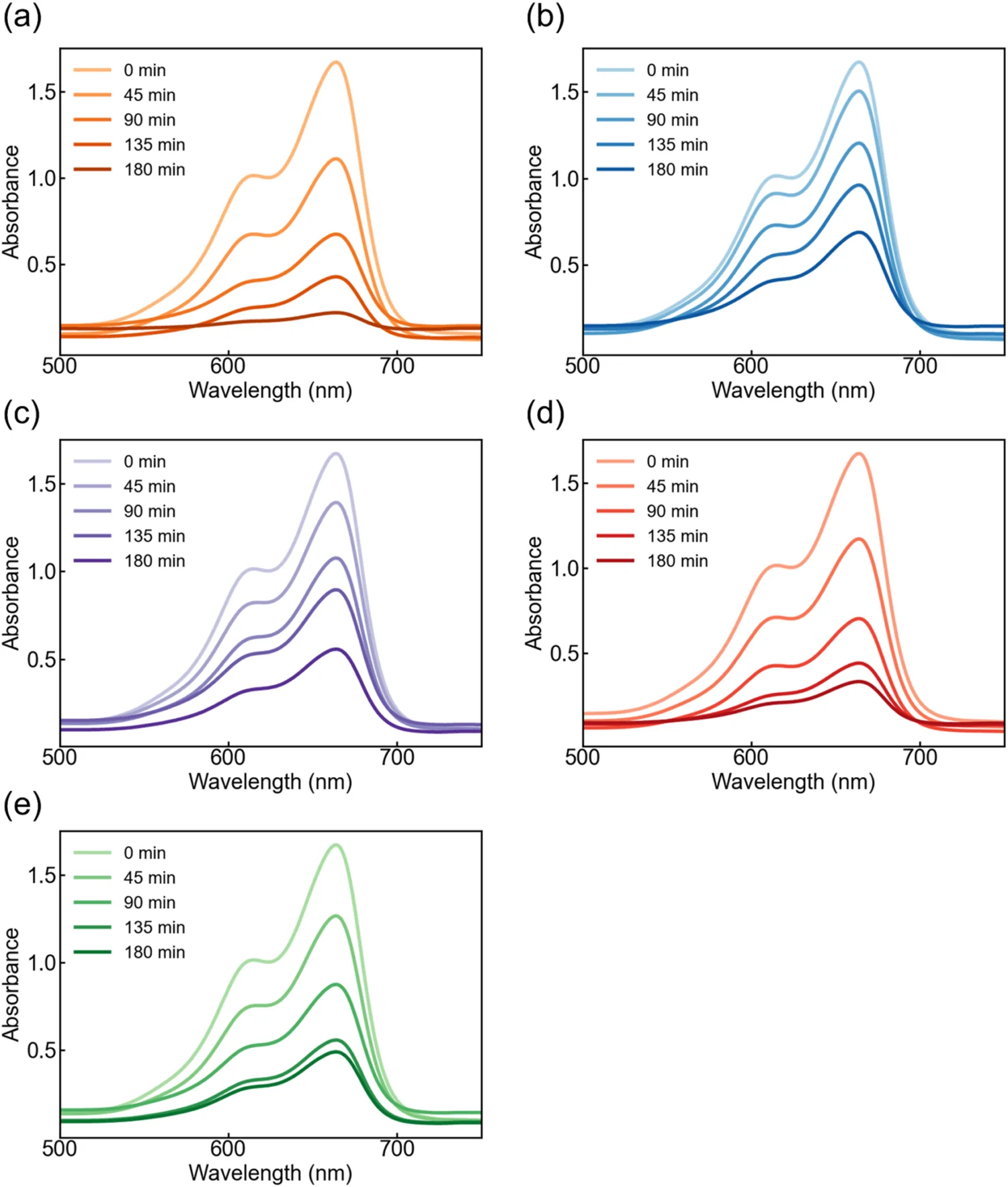
UV–vis absorbance spectra of methylene blue degradation over time for: (a) *Degussa P25* TiO_2_ powder, (b) pristine TiO_2_ film, and (c) TiO_2_–CeO_2_(1%) (d) TiO_2_–CeO_2_(2%), and (e) TiO_2_–CeO_2_(3%) under UV light irradiation.

Degradation over time was plotted as *C/C*_0_ against time (minutes) in Figure 5a, where *C* represents the MB concentration at a given time, and *C*_0_ the initial concentration. This normalized concentration plot allows a clear comparison of photocatalytic performances across samples. TiO_2_–CeO_2_ samples exhibited enhanced degradation compared to pristine TiO_2_, the TiO_2_–CeO_2_(2%) film achieving the highest degradation efficiency at approximately 80.1% after 180 min, followed by TiO_2_–CeO_2_(3%) films at 70.7%, while pure TiO_2_ and TiO_2_–CeO_2_ (1%) reached 58.9% and 66.7%, respectively. *Degussa* with the same catalyst loading reached 86.9% degradation after 180 min, comparable to the TiO_2_–CeO_2_(2%) film. It should be noted that the reported efficiencies refer to the decay of the characteristic MB absorption band at 664 nm (decolorization). Complete mineralization was not verified in this study and will require total organic carbon (TOC) analysis, although TiO_2_–based photocatalysts are documented to progressively mineralize MB to CO_2_, H_2_O, and inorganic ions under comparable UV conditions.^62^

**Figure 5:**
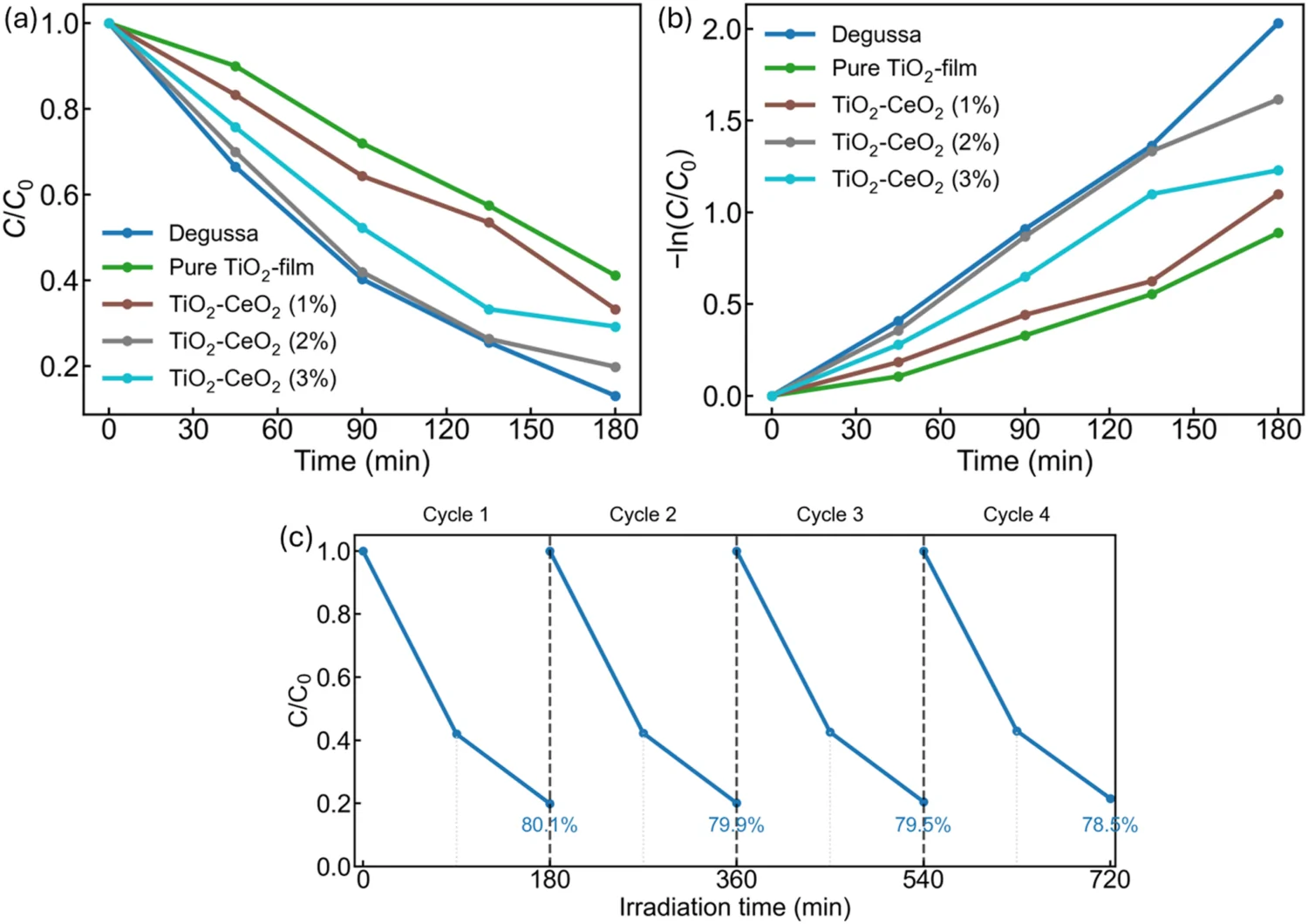
(a) Normalized MB concentration (*C*/*C*_0_) and (b) pseudo-first-order kinetics (−ln­(*C*/*C*_0_)) over time for TiO_2_ and TiO_2_–CeO_2_ films under UV light. (c) Four recycling runs of photocatalytic degradation of MB dye over the TiO_2_–CeO_2_ (2%) films catalyst.

To further analyze the degradation kinetics, we applied the pseudo-first-order model, plotting −ln­(*C*/*C*_0_) against time in Figure 5b. This model, common in photocatalysis studies, assumes the degradation rate is proportional to MB concentration.^63^ The plots are close to linear for all samples, which allows the determination of the apparent rate constants *k*. The TiO_2_–CeO_2_(2%) film has the highest rate constant, 9.1 × 10^–3^ min^–1^ (*R* ^2^ = 0.99), followed by the TiO_2_–CeO_2_(3%) (7.3 × 10^–3^ min^–1^, *R* ^2^ = 0.98), TiO_2_–CeO_2_(1%) (5.9 × 10^–3^ min^–1^, *R* ^2^ = 0.96) and pristine (4.9 × 10^–3^ min^–1^, *R* ^2^ = 0.97) films. *Degussa* gives 11.2 × 10^–3^ min^–1^ (*R* ^2^ = 0.99), consistent with its larger accessible surface area. The apparent rate constant therefore rises from pristine TiO_2_ to a maximum at 2 wt % CeO_2_ and decreases at 3 wt %, defining an optimal CeO_2_ loading.

Because the recyclability of immobilized photocatalysts is one of their principal advantages, the stability of the best-performing film was assessed over four consecutive degradation cycles (Figure 5c). The TiO_2_–CeO_2_(2%) film retained all its activity essentially: degradation at 180 min measured 80.1, 79.9, 79.5, and 78.5% over cycles one through four, corresponding to 98% retention, while the pseudo-first-order rate constant decreased only slightly, from 9.1 × 10^–3^ to 8.7 × 10^–3^ min^–1^ (95.5% retention). This near constant performance across repeated use confirms that the TiO_2_–CeO_2_ film functions as a stable, reusable photocatalyst and substantiates the practical case for the thin-film geometry.

The origin of this enhancement, however, cannot be resolved from the present data. The pristine and TiO_2_–CeO_2_(2%) films differ in microstructure as well as in composition (Section 3.1), and these contributions cannot be separated here. The effect is therefore attributed to the extended TiO_2_–CeO_2_ interface, without a specific mechanism being proposed. Further studies are necessary to determine the exact nature of enhanced performance and acting reactive oxygen species.

As noted above, the composite showed photocatalytic performance comparable to *Degussa P25*, despite the powder’s geometric advantage of exposing all particle faces to the solution. Direct measurement of the specific surface area by N_2_ physisorption (BET) does not apply to this film geometry, since the ∼0.3 mg of film on a 5 × 5 cm^2^ substrate lies below the sample quantity the technique requires.^64^ Surface characteristics were therefore assessed by direct electron microscopy (Section 3.1) and the comparison with *Degussa P25* rests on the geometric distinction between a dispersed powder accessible from all sides and a film exposing only one face.

Compared with previously reported TiO_2_ mixture systems (as summarized in Table 1 below), the photocatalytic rate constant and degradation efficiency of the TiO_2_–CeO_2_(2%) film are competitive, especially considering the ultra**-**thin film geometry, simpler synthesis via sol–gel, and moderate Ce content. These findings suggest that sol–gel TiO_2_–CeO_2_ thin films offer a promising, scalable alternative for photocatalytic applications in environmental remediation. Direct numerical comparison should nevertheless be made with caution, as the listed studies differ in light source and intensity, catalyst loading, dye concentration, and reactor geometry.

**Table 1:** Comparison of Photocatalytic Degradation Performance of Commercial and Previously Reported TiO_2_ Mixture Systems with the Thin Film Developed in This Work

catalyst type and form	impurity level (wt %)	synthesis method	light source	duration (h)	rate constant (min^‑1^)	efficiency (%)	refs
TiO_2_–CeO_2_(2 wt %) (thin film)	2	Sol–gel (dip-coat)	UV	3	9.1 × 10^–3^	∼80.1	this work
*Degussa P25* TiO_2_ (powder, benchmark)		Commercial	UV	3	11.2 × 10^–3^	∼86.9	this work
Ce-doped TiO_2_ (nanoparticles)	0.33	Sol–gel	Visible	8		90	^65^
Ce-TiO_2_–ZnO (powder)	1.0	Sol–gel	Visible	2		98	^66^
Ce-TiO_2_ (powder, visible light)	0.5	Hydrothermal	Visible	4	1.5 × 10^–3^	15	^67^
Ce-TiO_2_ (powder, UV light)	0.08	Hydrothermal	UV	4	5.0 × 10^–3^	∼93	^67^
CdS/TiO_2_/CeO_2_ on zeolite		Impregnation	Visible	2		99.9	^68^

## 4 Conclusions

In this work, ultrathin TiO_2_ films modified with CeO_2_ nanoparticles were prepared by a sol–gel dip coating method and evaluated as photocatalysts for methylene blue degradation under UVA irradiation. Elemental mapping showed cerium spatial distribution, consistent with a TiO_2_–CeO_2_ nanocomposite rather than with Ce ions incorporated into the TiO_2_ lattice, and XPS confirmed coexisting Ce^3+^/Ce^4+^ oxidation states. Among the investigated compositions, the TiO_2_–CeO_2_ film containing 2 wt % CeO_2_ showed the best, and overall competitive, photocatalytic performance under the applied UVA irradiation, reaching approximately 80% methylene blue degradation after 180 min and an apparent first-order rate constant of 9.1 × 10^–3^ min^–1^. This activity was substantially higher than that of pristine TiO_2_ and approached the performance of *Degussa P25* under comparable catalyst loading conditions, despite the limited exposed surface area of the thin film geometry.

The TiO_2_–CeO_2_(2%) film also proved to be durable, retaining 98.0% of its initial degradation efficiency with the first-order rate constant decreasing only slightly (from 9.1 to 8.7 × 10^–3^ min^–1^) over four consecutive cycles, confirming its operation as a stable, reusable photocatalyst. Overall, these results show that controlled CeO_2_ modification is an effective route to increase the photocatalytic activity of TiO_2_ thin films and support their potential as compact, recyclable photocatalysts for water purification applications.

## Supplementary Material



## Notes

X-ray diffraction pattern of the sol–gel-derived pristine TiO_2_ powder after heat treatment at 550 °C, indexed to anatase (Figure S1) (PDF)
