# Supplementary material for: Ultrathin TiO2–CeO2 Composite Films for Efficient Photocatalytic Methylene Blue Degradation
Source: ACS Omega. 2026 Sep 17;11(38):57895–905. doi: 10.1021/acsomega.6c07126 (PMC13629380; doi:10.1021/acsomega.6c07126)
Supplement: Supplementary file 1 [file ao6c07126_si_001.pdf]

Supporting Information

for

Ultra-Thin TiO_2-CeO_2 Composite Films for Efficient Photocatalytic Methylene Blue Degradation

Mahsa Amiri,[a] Majid Ahmadi,[b] Mahtab Salari Mehr,[a] Elyad Damerchi,[c] Khatereh Roohi,[d] Peyman Taheri,[d] Tanel Tätte,[a] Nadezda Kongi,[e] Alexander Vanetsev[a*]

[a] *Institute of Physics, University of Tartu, W. Ostwaldi 1, 50411 Tartu, Estonia*
[b] *Faculty of Science and Engineering, University of Groningen, 4 Nijenborgh, 9747 AG, Groningen, Netherlands*
[c] *Institute of Technology, University of Tartu, Nooruse 1, 50411 Tartu, Estonia*
[d] *Department of Materials Science and Engineering, Delft University of Technology, 2 Mekelweg, 2628 CD Delft, Netherlands*
[e] *Institute of Chemistry, University of Tartu, Ravila 14A, 50411 Tartu, Estonia*

*Corresponding author: Alexander Vanetsev (alexander.vanetsev@ut.ee)

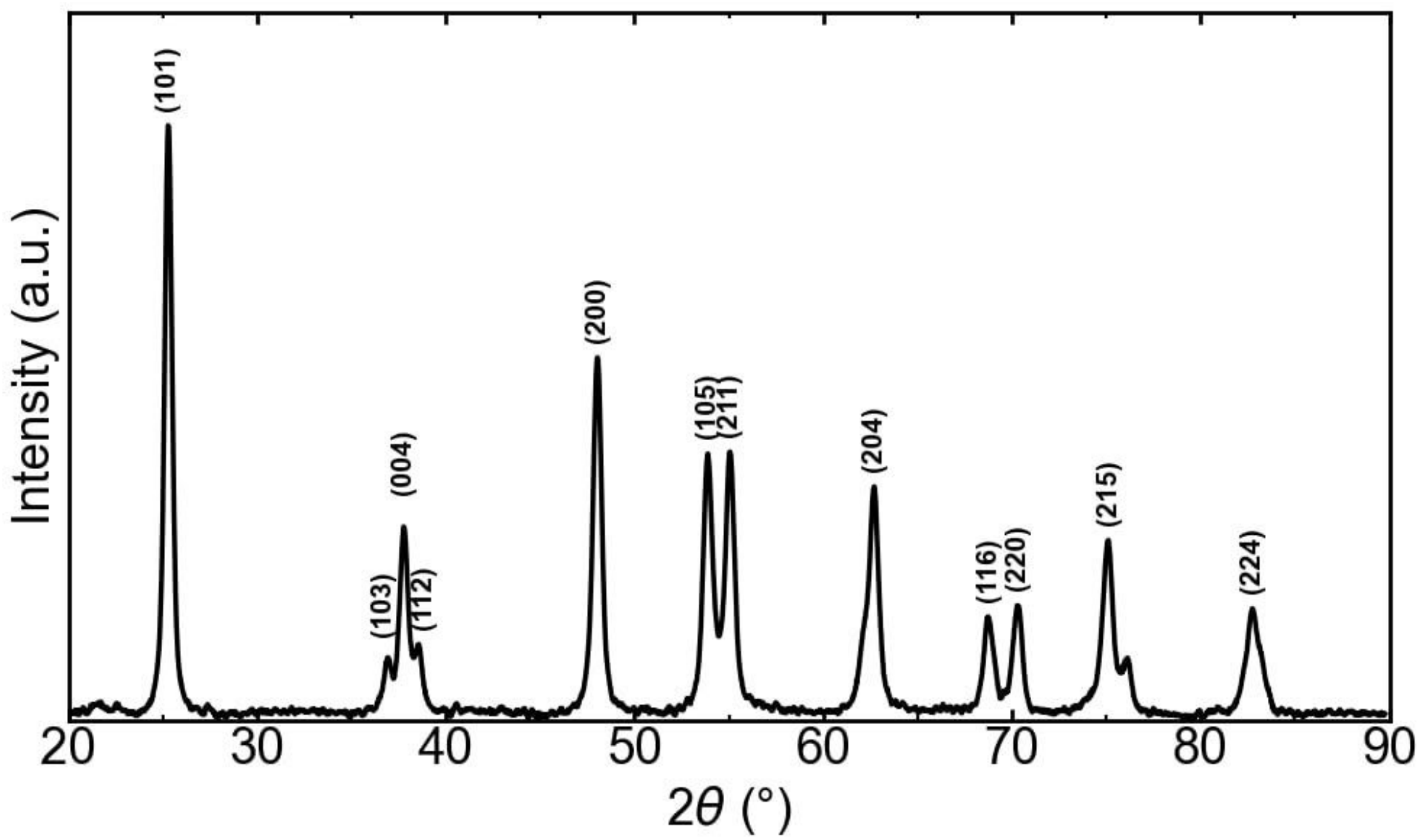

Figure S1. X-ray diffraction pattern of the sol–gel-derived pristine TiO_2 powder after heat treatment at 550 °C. All thirteen resolved reflections are indexed to anatase TiO_2. The pattern is shown after correction for a systematic specimen-displacement offset.
